# Variations in Quantity of Rain—Malaria

**Published:** 1840

**Authors:** 


### VARIATIONS IN THE QUANTITY OF RAIN—MALARIA.

After two years of drought, it is truly refreshing to have had a long *spell* of wet weather. The snows of last winter were deeper than they had been for years, and the months of March April and

May, brought down an unusual number of copious rains. The Ohio river which began to rise from its long and deep depression in February, continued in elevation throughout the next two months, and in May, overflowed its banks to a greater extent than had happened for the preceding six years, or, perhaps, since the great flood of 1832—when it reached a height not before witnessed since the first settlement of its valley.

This signal change in the humidity of the earth's surface, may be expected to exert an influence on the health of the people; and we earnestly invite our practical brethren, to be vigilant in their observations. Some localities may be more, others, less healthy, in the coming summer and autumn, than they were in the corresponding portions of the two preceding years, when the upper stratum of the earth was dry beyond all precedent. It would certainly be of deep professional interest to have all the facts touching this matter collected and recorded—especially if the work be executed in a proper spirit, that is, with a mind unwarped by any cherished theory of miasmatic diseases.

While on this subject, we cannot omit saying, that a heavy responsibility rests on the physicians of the Mississippi Valley, in regard to those summer and autumnal endemics, which are vaguely ascribed to malaria. We are very far from intimating, that this ascription should be compared to that, which in the dark ages of philosophy, explained the rise of water in a tube exhausted of air (nature's hatred of a vacuum;) but we *do* say, that the cause of autumnal fever, is not made known by the use of the terms malaria and miasma; and that no portion of the peopled earth, affords a better, a more promising field, for researches into the sources of autumnal disease, than that which stretches from the shores of the gulf, to those of the lakes. The man who shall discover the true, efficient cause of those diseases, would, in the immortality of his fame, be placed on the same page, with the discoverer of the circulation of the blood. D.

